# Supplementary material for: Productivity and Feed Quality Performance of Napier Grass (Cenchrus purpureus) Genotypes Growing under Different Soil Moisture Levels
Source: Plants (Basel). 2022 Sep 28;11(19):2549. doi: 10.3390/plants11192549 (PMC9572638; doi:10.3390/plants11192549)
Supplement: Supplementary file 1 [file plants-11-02549-s001.zip › plants-1917181-supplementary.pdf]

Supplementary materials

Table S1: Mean genotype values Napier grass accessions for Agro-morphology traits across harvests for Wet, MWS and SWS conditions

	PH			LL			LW			IL	ST	FvFm			PI			TN			TFW			TDW			LSR	WUE	
Genotype	Wet	MWS	SWS	Wet	MWS	SWS	Wet	MWS	SWS	Wet	Wet	Wet	MWS	SWS	Wet	MWS	SWS	Wet	MWS	SWS	Wet	MWS	SWS	Wet	MWS	SWS	Wet	MWS	SWS
1026	65.74	11.79	11.89	75.57	33.52	38.79	21.45	14.91	16.28	23.68	11.49	0.73	0.73	0.71	3.97	3.53	3.27	61.26	89.96	93.78	23.81	2.17	2.12	5.48	0.67	0.7	2.48	1.03	1.04
14355	72.78	19.03	18.32	85.34	48.87	50.97	25.49	19.76	22.43	24.15	14.8	0.76	0.73	0.71	6.45	3.29	3.36	61.07	94.61	109.19	60.43	6.35	7.03	14.37	1.68	1.82	2.13	2.97	3.36
14389	46.53	12.79	12.65	83.06	43.33	43.38	21.96	17.27	18.28	25.76	13.05	0.75	0.76	0.73	4.06	3.67	3.24	66.99	119.47	116	40.01	5.67	5.58	7.96	1.66	1.42	9.12	2.33	2.3
14982	46.74	14.59	13.54	82.84	47.49	47.65	21.67	16.65	16.55	31.8	45.59	0.75	0.74	0.72	4.21	3.41	3.18	84.44	157.84	163.59	41.95	7.5	7.78	8.38	2.25	1.91	9.51	3.07	3.01
14983	65.76	11.72	11.44	84.25	36.98	38.94	23.98	14.47	15.55	22.14	13.81	0.74	0.74	0.73	5.09	3.71	3.32	129.11	277.53	277.77	61.83	6.87	7.08	12.77	1.83	1.71	3.42	2.81	2.88
14984	54.67	15.25	14.6	85.86	45.62	45.64	30.89	20.71	20.98	26.83	15.95	0.75	0.73	0.73	4.66	3.23	3.3	48.38	117.24	114.31	49.36	6.43	6.04	11.62	1.92	1.63	5.57	2.74	2.6
15357	70.18	17.13	15.76	85.96	48.35	47.01	25.51	19.2	19.82	24.2	14.98	0.74	0.74	0.72	4.42	3.83	3.91	55.12	102.27	101.63	63.16	6.35	5.4	14.67	1.85	1.35	2.08	2.44	2.16
15743	68.04	12.41	11.98	82.47	42.88	43.89	26.93	16.61	17.54	24.41	15.21	0.76	0.76	0.74	4.64	3.99	3.81	42	108.53	96.76	46.34	5.31	5	11.57	1.4	1.26	2.41	2.1	2.02
16621	45.37	6.98	6.99	28.25	14.09	15.72	12.72	10.01	10.37	13.78	5.15	0.77	0.77	0.75	5.6	4.77	4.37	24.32	28.88	21.61	2.06	1.37	1.28	0.63	0.32	0.3	3	0.64	0.58
16782	51.49	11.35	12.33	80.22	35.98	40.83	24.45	17.22	18.25	21.61	13.95	0.74	0.74	0.73	4.23	4.6	3.94	66.74	136.72	131.13	37.61	2.87	3.69	9.68	0.79	0.93	4.86	1.29	1.5
16783	65.17	13.88	12.61	79.97	47.83	43.14	27.15	18.65	18.93	24.03	15.67	0.77	0.71	0.7	5.5	3.08	2.8	40.1	116.66	109.05	46.75	5.71	4.85	11.66	1.64	1.24	2.61	2.15	1.81
16784	55.03	12.51	11.87	84.03	40.46	43.11	25.53	16.85	17.94	22.97	13.78	0.73	0.72	0.7	4.18	4.15	2.88	83.91	146.2	158.52	44.58	4.3	4.78	9.63	1.15	1.29	4.92	1.8	2.05
16785	40.94	15.04	14.76	82.45	48.5	49.41	26.96	20.84	22.05	25.71	13.61	0.73	0.73	0.71	3.85	2.28	2.21	73.03	118.5	124.5	29.31	5.91	6.15	7.46	1.66	1.83	9.38	2.59	2.95
16786	52.64	15.28	14.99	88.36	50.31	47.54	26.72	21.26	21.18	28.46	15.65	0.74	0.73	0.72	4.52	2.92	2.63	57.81	113.53	108.98	43.09	5.92	5.22	9.26	1.72	1.38	7.09	2.44	2.27
16787	36.28	14.67	13.77	75.27	49.15	48.68	23.59	20.59	22.32	25.84	11.73	0.73	0.74	0.72	4.22	3.11	3.06	55.53	83.63	69.53	25.24	4.45	3.48	6.24	1.37	1.01	10.57	1.66	1.51
16788	60.79	13.96	12.99	80.71	43.64	43.85	29.99	18.19	18.89	22.3	14.85	0.76	0.74	0.72	4.78	3.8	3.22	39.18	108.53	95.6	36.68	5.05	4.33	8.29	1.42	1.08	3.1	1.93	1.7
16789	53.64	16.79	16.07	86.28	52.58	50.7	30.87	22.26	22.57	31.4	16.02	0.75	0.73	0.72	4.3	3.04	3	47.34	100.88	101.33	46.84	6.5	6.46	10.6	1.96	1.84	5.25	3	3.06
16790	53.3	6.79	6.15	58.31	24.37	22.9	17.8	11.09	11.25	22.8	10.31	0.73	0.73	0.72	3.77	4.65	3.27	60.95	130.92	101.86	16.27	1.49	0.78	3.35	0.4	0.29	2.61	0.6	0.43
16791	65.59	14.28	13.75	81.89	44.47	46.8	26.55	17.94	19.63	21.74	15.49	0.73	0.75	0.73	3.88	4.17	4.04	87.29	220.36	234.44	81.64	7.93	8.59	20.38	2.17	2.21	2.96	3.51	3.82
16792	48.92	15.45	15.26	85.54	49.37	49.1	29.66	21.27	21.57	26.59	15.62	0.75	0.74	0.73	4.82	3	2.89	44.62	106.25	100.29	48.07	6.65	6.8	11.12	1.92	1.82	4.89	3.1	3.18
16793	56.64	15.89	15.25	86.97	46.6	43.25	29.5	23.17	22.84	23.43	14.85	0.73	0.74	0.74	4.14	3.66	4.01	47.41	97.72	90.55	31.1	4.82	4.06	6.94	1.43	1.16	3.66	2.16	2.01
16794	49.36	12.34	11.6	78.86	38.35	37.4	24.56	16.78	17.5	29.49	13.45	0.78	0.75	0.75	6.19	4.19	4.16	93.35	184.55	195.09	43.01	5.72	5.49	9.23	1.65	1.58	6.4	2.64	2.81
16795	50.67	16.17	15.84	85.29	50.6	50.25	29.28	21.51	21.88	24.9	15.19	0.74	0.74	0.72	5	3.24	3.1	44.69	104.95	102.11	47.98	8.17	7.87	11.43	2.43	2.2	6.62	3.38	3.42
16796	49.92	9.56	9.58	73.4	35.77	35.48	24.22	15.66	15.88	29.27	11.99	0.75	0.74	0.74	4.1	4	3.79	57.59	117.58	114.57	28.89	2.81	2.42	6.06	0.82	0.69	4.57	1.29	1.14
16797	37.8	8.78	8.62	60.9	30.87	34.44	19.37	14.08	15.78	27.15	9.88	0.73	0.76	0.75	4.66	4.21	3.82	29.76	55.33	55.93	8.93	1.15	1.36	1.77	0.29	0.33	8.41	0.46	0.52

16798	51.71	16.29	15.98	86.5	53.62	50.28	30.34	21.39	21.58	22.35	17.05	0.73	0.74	0.74	4.77	3.24	3.23	44.04	108.54	113.07	42.61	6.07	6.28	8.76	1.82	1.84	6.01	2.82	2.95
16799	54.84	12.32	11.74	80.44	42.56	41.85	23.43	16.46	16.63	31.39	12.78	0.72	0.71	0.7	3.59	2.45	2.2	55.55	111.76	102.35	36.26	4.06	3.28	7.18	1.11	0.85	5.03	1.58	1.37
16800	53.29	15.98	16.05	85.62	50.02	50.55	30.98	20.96	21.8	28.23	15.84	0.74	0.72	0.7	4.44	2.99	2.71	44.98	95.92	94.7	41.13	6.24	6.07	9.23	1.82	1.61	7.5	2.66	2.63
16801	53.42	16.13	15.14	82.15	47.59	45.46	28.77	19.72	20.67	26.15	15.76	0.74	0.72	0.71	4.17	2.68	2.92	44.25	82.94	82.1	38.95	5.31	5.25	8.31	1.62	1.47	5.35	2.53	2.48
16802	67.14	15.99	15.6	83.47	45.19	46.66	26.88	19.39	20.61	23.95	15.52	0.75	0.72	0.73	4.74	3.57	3.72	82.31	204.39	230.51	67.55	7.09	8.27	16.34	1.99	2.19	2.38	3.62	4.1
16803	38.94	14.77	14.22	81.16	50.22	50.1	25.53	20.53	20.46	27.23	13.27	0.75	0.72	0.7	4.1	2.84	2.8	72.12	117.63	100.09	32.69	8.82	6.97	7.38	2.65	1.91	7.4	3.55	2.91
16804	44.03	14.16	13.03	77.96	45.13	45.83	24.73	19.32	19.91	28.17	13.33	0.73	0.71	0.7	4.15	3.06	3.12	60.86	113.26	101.61	39.93	5.25	4.29	8.42	1.39	1.04	6.69	1.87	1.66
16805	30.4	7.62	8.32	55.61	27.07	33.88	20.23	15.28	17.25	24.77	9.5	0.73	0.73	0.72	3.66	3.27	4.19	24.84	42.95	37.81	6.13	0.89	1.06	1.41	0.24	0.32	9.33	0.42	0.48
16806	43.12	14.36	13.89	79.13	48.13	47.77	25.44	20.69	21.78	23.71	13.53	0.74	0.73	0.71	4.46	2.83	2.73	77.51	130.7	115.53	37.06	6.34	5.23	8.12	1.91	1.49	8.58	2.61	2.33
16807	50.55	9.91	10.03	84.89	35.04	36.87	26.63	16.47	16.89	25.63	14.67	0.72	0.72	0.7	3.28	3.42	3.25	87.69	171.06	157.23	53.74	5.39	5.8	13	1.16	1.26	8.88	1.82	1.99
16808	57.86	10.03	10.04	73.15	36.02	38.12	27.81	16.62	17.93	20.8	13.28	0.75	0.76	0.74	4.65	4.26	4.09	56.72	132.97	118.23	37.72	2.9	2.97	8.9	0.89	0.81	4.64	1.26	1.21
16809	57.41	9.74	9.73	74.4	37.53	40.63	28.98	16.99	17.52	20.85	13.72	0.74	0.74	0.72	3.68	3.64	3.1	73.55	184.71	148.77	41.87	4.5	3.24	9.64	1.35	0.97	3.2	1.73	1.34
16810	54.19	9.27	10.35	69.8	35.93	39.64	26.32	16.06	17.26	21.57	12.33	0.72	0.74	0.73	3.37	3.84	3.63	50.39	111.27	113.31	26.96	1.95	2.11	6.53	0.55	0.62	5.45	1.09	1.17
16811	58.27	13.76	13.7	88.81	43.69	43.12	26.83	18.36	19.84	26.07	14.12	0.72	0.73	0.72	3.28	3.33	3.15	86.74	185.42	171.76	55.39	8.46	7.24	11.26	2.46	1.96	7.69	3.26	2.96
16812	57.49	11.41	11.68	82.51	38.21	41.85	27.49	18.3	19.58	21.09	14.92	0.76	0.74	0.73	5.15	4.47	4.52	49.43	105.6	101.75	44.46	3.18	3.37	9.23	0.78	0.83	3.98	1.21	1.33
16813	39.56	7.97	8.95	66.45	30.5	37.63	25.03	17.1	19.03	17.1	12.1	0.72	0.74	0.72	4.21	4.33	3.86	97.4	157.21	177.1	29.62	3.21	4.93	6.56	0.81	1.23	6.65	1.57	2.03
16814	69.58	14.21	13.5	86	45.04	46.04	27.34	17.63	19.11	20.92	14.9	0.74	0.74	0.73	3.37	4.66	4.28	86.31	179.46	176.61	58.3	5.99	6.38	12.57	1.65	1.61	2.72	2.56	2.75
16815	61.01	11.19	12.03	83.26	40.76	41.15	28.92	17.45	19.57	22.43	15.36	0.74	0.73	0.71	4.34	3.56	3.45	44.97	101.42	87.47	50.61	4.26	4.18	10.8	1.07	1.04	4.66	1.51	1.62
16816	40.85	10.04	9.87	68.91	29.1	29.81	24.94	13.54	14.35	24.71	11.28	0.74	0.74	0.74	5.07	4.44	4.45	57.59	156.84	148.71	30.26	2.73	2.69	6.78	0.74	0.65	6.52	1.07	1.02
16817	64.53	15.68	15.11	84.57	44.87	47.9	27.03	17.13	19.08	21.26	13.99	0.75	0.74	0.72	4.53	3.62	2.97	59.39	121.38	129.02	42.64	5.82	5.79	10.19	1.42	1.45	2.24	2.06	2.27
16818	56.87	8.96	9.39	69.6	32.66	32.25	28.39	16.11	16.5	19.55	12.62	0.74	0.73	0.72	4.2	2.97	2.94	54.34	128.28	141.51	38.11	1.9	2.39	7.79	0.61	0.68	4.62	1.06	1.16
16819	62.74	16.47	16.85	87.83	46.55	51.18	30.06	21.02	22.91	23.92	16.27	0.74	0.75	0.74	3.96	3.92	3.73	49.7	133.02	130.76	80.83	10.14	9.7	18.33	2.79	2.54	2.8	3.94	4.12
16821	56.71	9.12	9.26	70.54	32.75	35.48	26.7	16.04	16.81	20.04	12.6	0.76	0.75	0.73	4.41	3.84	3.52	59.61	128.76	134.12	32.94	2.5	2.58	7.27	0.73	0.72	3.34	1.19	1.25
16822	57.58	8.67	9.19	74.74	33.6	37.06	29.32	16.24	16.94	24.29	16.03	0.72	0.72	0.71	3.36	3.76	3.29	72.16	148.11	149.39	42.23	2.68	3.16	8.97	0.76	0.85	4.27	1.15	1.22
16834	41.99	7.14	8.32	68.05	24.4	32.34	20.25	12.25	14.34	26.91	10.54	0.74	0.74	0.72	4.8	4.05	3.38	44.86	83.74	89.36	24.72	1.9	1.92	4.47	0.43	0.53	6.07	0.84	0.95
16835	58.14	10.99	10.36	78.85	41.84	38.72	26.33	17.55	17.33	22.25	13.49	0.75	0.73	0.72	4.52	3.4	3.26	44.5	82.14	75.56	23.65	3	2.4	5.47	0.8	0.71	3.66	1.08	1
16836	40.87	11.72	10.94	77.75	36.41	35.46	24.92	18.45	19.31	27.29	12.38	0.73	0.74	0.71	3.94	2.98	2.52	53.74	82.95	72.17	23.05	2.88	2.66	6.15	0.85	0.8	8.98	1.26	1.28
16837	54.8	13.61	12.12	82.81	45.81	42.03	23.28	17.9	18.32	27.3	13.02	0.74	0.72	0.71	4.77	2.77	2.6	62.92	125.53	109.61	35.1	5.98	3.99	7.52	1.51	0.93	5.59	1.88	1.46
16838	49.79	9.6	9.9	78.53	33.02	38.62	22.5	14.07	15.69	28.81	12.25	0.73	0.73	0.72	3.72	4.26	3.9	73.02	119.92	113.83	26.84	2.24	3.27	5.85	0.61	0.81	5.95	1.07	1.33
16839	69.86	14.02	13.05	80.99	42.23	39.6	23.47	15.32	15.1	21.08	14.16	0.74	0.71	0.71	4.05	3.43	3.33	134.95	289.45	275.89	65.79	9.45	8.92	13.42	2.55	2.29	2.58	3.58	3.49

16840	55.98	15.81	13.44	85.44	52.87	48.85	24.48	18.27	18.39	26.02	13.03	0.73	0.73	0.72	4.14	3.09	3.07	67.85	136.82	130.79	40.93	5.66	4.54	8.6	1.55	1.09	5.98	2.11	1.82
16902	40.67	12.05	11.96	80.14	42.16	45.36	23.15	16.99	18.49	26.05	12.75	0.76	0.75	0.74	5.19	3.86	4.06	53.64	89.18	89.03	32.55	4.21	4.18	6.34	1.21	1.21	12.21	2.23	2.33
18438	55.83	12.99	12.47	84.69	44.79	43.99	29.69	19.49	20.29	20.79	15.3	0.74	0.73	0.75	6.03	4.14	4.49	71.07	159.71	160.09	57.74	5.02	4.62	12.6	1.38	1.14	3.54	2.23	2.09
18448	54.71	11.76	10.92	84.62	40.11	38.36	25.91	17.82	18.43	24.19	14.95	0.76	0.74	0.74	5.35	4.39	4.27	61.54	142.4	134.36	49.57	4.95	4.21	11.85	1.31	1.05	13.99	1.9	1.71
18662	33.31	5.73	5.37	36.49	18.88	20.11	15.33	11.31	11.85	16.82	7.16	0.77	0.74	0.72	4.84	3.57	2.93	18.68	42.23	38.74	2.94	0.98	0.79	0.63	0.27	0.23	2.64	0.55	0.67
BAGCE 100	76.39	14.25	14.27	84.55	50.51	52.25	29.41	19.57	20.16	24.18	13.35	0.74	0.71	0.71	3.04	2.77	3.08	83.1	207.72	220.75	70.91	6.43	8.54	16.15	1.86	2.27	2.45	2.89	3.52
BAGCE 17	55.57	10.03	9.8	85.82	34.86	36.6	25.01	16.93	17.41	23.7	15.25	0.74	0.71	0.71	4.92	3.32	3.37	70.03	161.56	157.73	50.64	4.19	5.08	11.41	1.01	1.15	12.23	1.64	1.87
BAGCE 30	70.76	12.84	12.7	88.82	49.9	50	28.71	19.88	20.58	24.39	14.46	0.74	0.71	0.7	4.88	3.25	3.05	101.48	243.77	262.27	79.57	8.92	8.85	17.36	2.5	2.22	2.93	3.72	3.61
BAGCE 34	60.33	13.16	12.98	84.34	47.73	49.33	24.8	18.32	19.85	28.36	13.91	0.73	0.76	0.75	3.76	4.6	4.51	84.48	231.97	235.19	70.65	7	7.48	15.44	1.84	1.79	6.08	3.08	3.26
BAGCE 53	69.65	11.95	11.79	79.46	37.38	41.78	28.51	16.29	18.6	23.97	13.39	0.72	0.73	0.72	3.4	3.16	2.97	85.72	193.49	172	60.59	5.54	5.31	12.58	1.54	1.31	3.18	2	1.88
BAGCE 81	57.32	11.84	11.23	81.98	36.71	38.31	27.6	18.21	19.13	22.75	13.64	0.72	0.75	0.73	3.77	4.99	3.77	62.6	174.92	151.07	50.48	5.36	4.98	11.37	1.48	1.38	5.37	1.94	1.88
BAGCE 86	62.43	10.04	9.99	75.41	33.25	34.47	22.77	14.47	15.67	23.07	12.87	0.73	0.77	0.75	4.2	4.01	3.64	98.37	237.27	214.52	50.43	6.26	5.76	10.5	1.56	1.38	3.43	1.96	1.95
BAGCE 93	62.88	12.46	12.47	84.05	45.26	47.94	27.14	17.78	19.79	23.44	14.64	0.75	0.76	0.73	4.66	4.27	3.51	80.01	219.55	215.61	67.03	8.58	8.64	14.98	2.29	2.15	3.22	3.32	3.35
BAGCE 97	56.29	12.89	11.87	80.56	46.23	47.16	27.94	19.55	21.83	25.74	14.11	0.73	0.73	0.73	3.64	3.28	3.32	62.27	165.47	172.8	54.95	7.37	7.73	12.35	1.92	1.98	4.11	3.19	3.49
CNPGL 00-1-1	61.95	12.57	11.39	84.52	40.15	37.4	25.64	17.54	17.63	21.6	15.8	0.74	0.74	0.73	4.13	4.34	4.18	92.61	225.77	184.96	61.61	6.9	5.36	14.26	1.79	1.3	4.25	2.43	1.98
CNPGL 92-133-3	57.94	14.33	14.62	87.81	45.64	49.16	28.65	22.28	23.37	26.67	14.09	0.74	0.71	0.71	4.69	3.46	3.78	38.89	68.49	77.57	39.23	4.86	5.2	9.09	1.21	1.2	6.15	1.73	1.87
CNPGL 92-198-7	71.51	13.39	12.21	81.93	46.66	43.15	29.53	19.84	19.79	23.59	14.25	0.75	0.75	0.75	3.96	4.85	4.66	81.26	183.56	171.04	70.12	8.05	7.07	14.94	2.06	1.73	2.54	2.95	2.79
CNPGL 92-56-2	70.87	13.49	13.3	86.75	45.05	44.63	28.63	19.87	20.4	23.5	14.85	0.74	0.74	0.71	3.68	2.91	2.55	46.09	112.98	108.79	59.41	6.94	6.32	13.77	1.92	1.79	3.36	2.55	2.6
CNPGL 92-66-3	61.1	16.25	16.64	89.33	52.06	54.8	29.76	21.06	23.12	25.14	16.34	0.72	0.74	0.73	4.4	4.23	4.01	85.65	169.93	179.07	53.75	8.24	8.8	13.57	2.36	2.39	4.15	3.73	3.98
CNPGL 9279-2	52.08	15.75	14.82	92.68	53.46	51.58	29.84	23.37	23.5	28.59	15.51	0.75	0.73	0.72	3.9	3.56	3.28	38.55	81.78	70.82	35.69	6.47	5.44	9.4	1.87	1.32	4.91	2.35	1.97
CNPGL 93 -37-5	66.16	14.93	14.06	83.69	43.73	44.32	28.89	19.17	20.9	22.98	15.41	0.74	0.72	0.71	3.72	3.08	3.21	94.45	210.79	205.8	72.5	9.19	9.88	16.35	2.36	2.42	2	3.64	3.97
CNPGL 93-01-1	47.23	15.78	14.06	79.48	51.45	49.96	27.37	22.46	22.97	24.71	14.85	0.75	0.75	0.73	5.18	4.17	3.65	43.28	102.62	87.62	31.53	7.07	5.81	6.97	2.12	1.38	9.17	2.45	1.84
CNPGL 93-04-2	60.39	11.89	11.99	82.46	40.69	43.06	27.04	19.51	20.46	22.03	14.16	0.71	0.75	0.73	4.69	4.07	3.6	93.37	198	189.26	55.11	4.93	5.84	11.86	1.2	1.44	3.32	2.16	2.41
CNPGL 93-18-2	56.21	12.92	12	88.52	47.45	47.98	28.38	19.14	20.53	21.25	16.33	0.75	0.75	0.72	4.42	4.28	3.65	41.55	92.54	73.82	35.12	5.75	4.78	7.31	1.43	1.03	3.29	1.79	1.49
CNPGL 94-13-1	61.14	15.04	14.5	86.89	44.4	46	30.87	19.63	20.36	23.95	16.85	0.74	0.74	0.73	5.04	4.39	4.16	42.45	91.55	99.51	50.25	5.89	5.99	11.36	1.54	1.41	5.01	2.35	2.35
CNPGL 96-21-1	58.25	14.48	13.8	73.17	45.22	46.55	24.48	19.33	20.11	24.26	13.61	0.71	0.74	0.72	3.93	3.69	3.04	71.83	128.51	131.17	40.15	5.05	5.16	8.33	1.29	1.22	3.55	2.21	2.25
CNPGL 96-23-1	54.87	10.72	10.62	71.33	31.32	34.95	23.79	16.9	17.24	21.72	12.54	0.74	0.75	0.73	3.3	3.82	3.7	55.68	122.07	113.68	28.31	2.88	2.89	6.96	0.78	0.82	4.44	1.3	1.39
CNPGL 96-27-3	54.46	14.76	13.69	81.83	44.61	42.9	27.33	18.05	18.53	22	14.38	0.72	0.75	0.73	5.46	4.31	4.18	71.54	144.53	159.25	48.41	6.1	5.43	9.8	1.61	1.32	3.66	2.22	2.16
PIONEIRO	68.05	11.08	10.35	73.94	38.98	37.74	24.42	15.83	16.44	21.73	12.8	0.74	0.76	0.76	4.52	4.07	3.97	96.8	212.59	236.61	45.23	6.45	5.85	10.02	1.61	1.45	3.7	2.46	2.43
LSD 5%	0.71	0.99	0.99	0.69	3.24	3,24	0.19	1.21	1.21	4.02	0.14	0.02	0.01	0.01	0.86	0.51	0.51	3.63	4.76	4.77	1.21	0.48	0.48	0.43	0.17	0.17	2.34	0.2	0.2

Table S2: Mean genotype values Napier grass accessions for feed quality traits across harvests for Wet, MWS and SWS conditions

	NDF			ADF			ADL			OM			CP			IVOMD			Me		
Genotypes	**Wet**	**MWS**	**SWS**	**Wet**	**MWS**	**SWS**	**Wet**	**MWS**	**SWS**	**Wet**	**MWS**	**SWS**	**Wet**	**MWS**	**SWS**	**Wet**	**MWS**	**SWS**	**Wet**	**MWS**	**SWS**
1026	**68.41**	**64.9**	**62.21**	**41.26**	**35.31**	**30.93**	**3.94**	**2.69**	**2.38**	**83.33**	**83.39**	**83.12**	**11.32**	**11.39**	**16.14**	**54.72**	**57.28**	**60.39**	**7.63**	**8.07**	**8.35**
14355	**68.09**	**64.66**	**63.81**	**41.35**	**36.8**	**32.86**	**3.84**	**2.79**	**2.65**	**83.32**	**82.68**	**83.71**	**12.22**	**11.04**	**14.41**	**55.03**	**55.99**	**59.82**	**7.66**	**7.88**	**8.38**
14389	**67.01**	**62.81**	**61.79**	**41.12**	**38.12**	**35.92**	**3.85**	**2.91**	**2.83**	**82.26**	**81.52**	**80.83**	**12.83**	**11.53**	**13.81**	**55.53**	**56.47**	**58.39**	**7.65**	**7.92**	**8.06**
14982	**66.79**	**62.97**	**62.24**	**40.83**	**37.94**	**36.41**	**3.83**	**2.95**	**2.82**	**82.1**	**81.39**	**81.54**	**12.79**	**11.78**	**13.14**	**55.56**	**56.62**	**57.68**	**7.65**	**7.91**	**7.99**
14983	**67.48**	**62.35**	**61.76**	**41.51**	**36.98**	**32.77**	**3.97**	**2.92**	**2.77**	**82.58**	**80.82**	**82.29**	**12.71**	**13.72**	**16.76**	**55.63**	**57.3**	**60.12**	**7.7**	**7.95**	**8.32**
14984	**67.34**	**64.05**	**63.1**	**41.93**	**37.76**	**35.42**	**4.02**	**2.74**	**2.69**	**82.77**	**82.15**	**82.07**	**12.34**	**9.88**	**12.31**	**55.12**	**55.75**	**57.86**	**7.64**	**7.87**	**8.09**
15357	**68.84**	**65.71**	**64.56**	**41.47**	**36.36**	**33.6**	**3.86**	**2.77**	**2.64**	**83.82**	**84.16**	**84.05**	**11.74**	**10.78**	**13.43**	**55.39**	**56.25**	**58.12**	**7.73**	**7.98**	**8.15**
15743	**69.35**	**63.67**	**63.25**	**43.25**	**38.42**	**36.91**	**3.94**	**2.81**	**2.73**	**83.15**	**81.9**	**81.21**	**11.21**	**10.22**	**11.78**	**54.41**	**56.32**	**57.27**	**7.57**	**7.91**	**7.94**
16621	**69.67**	**67.65**	**66.56**	**39.77**	**39.55**	**36.03**	**4.21**	**3.34**	**3.29**	**85.76**	**84.34**	**86.21**	**12.93**	**11.83**	**14.14**	**56.15**	**54.75**	**57.17**	**7.83**	**7.68**	**7.99**
16782	**67.35**	**62.39**	**62.12**	**40.75**	**36.77**	**35.19**	**3.88**	**2.95**	**2.8**	**82.96**	**81.09**	**82.7**	**11.83**	**10.81**	**12.59**	**55.29**	**55.32**	**56.4**	**7.67**	**7.77**	**7.84**
16783	**68.9**	**64.23**	**63.82**	**43.12**	**39.28**	**35.57**	**3.93**	**2.83**	**2.8**	**82.69**	**81.04**	**82.13**	**11.05**	**9.14**	**13.12**	**54.29**	**55.09**	**58.55**	**7.57**	**7.71**	**8.13**
16784	**66.39**	**63.07**	**61.62**	**40.92**	**37.46**	**34.15**	**3.83**	**3**	**2.81**	**81.97**	**80.71**	**80.77**	**12.43**	**11.74**	**15.98**	**55.69**	**56.7**	**59.71**	**7.69**	**7.88**	**8.18**
16785	**67.65**	**66.28**	**63.78**	**41.17**	**38.12**	**34.43**	**3.8**	**2.67**	**2.44**	**82.48**	**83.29**	**82.6**	**11.05**	**7.56**	**11.7**	**54.7**	**54.62**	**57.63**	**7.62**	**7.79**	**8.08**
16786	**69.12**	**64.85**	**63.34**	**42.68**	**37.73**	**34.34**	**4.18**	**2.69**	**2.63**	**83.55**	**82.33**	**83.09**	**11.77**	**9.35**	**12.25**	**54.08**	**54.58**	**57.5**	**7.54**	**7.71**	**8.06**
16787	**66.53**	**65.12**	**63.45**	**40.12**	**38.11**	**34.05**	**3.73**	**2.57**	**2.47**	**82.37**	**82.82**	**83**	**12.6**	**9.63**	**14.42**	**55.74**	**54.93**	**59.22**	**7.71**	**7.78**	**8.25**
16788	**67.51**	**64.43**	**63.35**	**41.81**	**38.47**	**34.88**	**3.91**	**2.82**	**2.75**	**82.44**	**81.84**	**82.41**	**11.81**	**10.41**	**13.99**	**54.92**	**55.95**	**59.04**	**7.62**	**7.85**	**8.17**
16789	**67.95**	**64.19**	**63.23**	**41.98**	**38.45**	**35.68**	**4.03**	**2.83**	**2.73**	**83.03**	**81.5**	**81.87**	**11.87**	**8.79**	**11.75**	**54.76**	**55.05**	**57.53**	**7.63**	**7.76**	**8.03**
16790	**65.97**	**61.79**	**62.8**	**39.95**	**37.68**	**33.93**	**3.54**	**2.8**	**2.51**	**81.7**	**78.82**	**81.68**	**12.64**	**10.79**	**13.92**	**55.64**	**56.26**	**59.14**	**7.69**	**7.82**	**8.25**
16791	**68.29**	**63.66**	**62.45**	**41.8**	**36.78**	**33.87**	**3.78**	**2.56**	**2.44**	**82.53**	**81.3**	**81.6**	**11.9**	**10.68**	**14.35**	**55.14**	**56.72**	**59.47**	**7.67**	**7.97**	**8.25**
16792	**68.58**	**64.59**	**62.98**	**42.41**	**39.56**	**37.1**	**3.97**	**2.8**	**2.75**	**83.09**	**81.7**	**81.25**	**11.15**	**7.93**	**10.86**	**54.26**	**54.07**	**56.36**	**7.57**	**7.65**	**7.87**
16793	**68.1**	**65.63**	**64.87**	**41.06**	**36.99**	**34.69**	**3.89**	**2.66**	**2.59**	**83.18**	**82.65**	**82.72**	**12.08**	**9.33**	**11.6**	**55**	**54.71**	**56.83**	**7.61**	**7.75**	**7.99**
16794	**66.9**	**63.48**	**62.99**	**41.89**	**37.94**	**33.72**	**4.2**	**2.93**	**2.74**	**82.09**	**81.81**	**83.07**	**12.69**	**11.13**	**16.08**	**54.55**	**55.68**	**59.32**	**7.52**	**7.8**	**8.23**
16795	**67.76**	**64.69**	**63.29**	**42.01**	**38.99**	**36.48**	**3.99**	**2.8**	**2.69**	**82.77**	**81.97**	**81.68**	**11.73**	**9.61**	**11.92**	**54.8**	**55.59**	**57.59**	**7.6**	**7.84**	**8.04**
16796	**68.65**	**62.77**	**62.88**	**41.23**	**38.39**	**34.98**	**3.86**	**2.77**	**2.71**	**82.43**	**80.15**	**81.94**	**11.97**	**10.57**	**13.71**	**54.83**	**55.61**	**58.53**	**7.6**	**7.75**	**8.13**
16797	**66.82**	**64.37**	**63.91**	**39.17**	**36.23**	**34.69**	**3.58**	**2.61**	**2.62**	**82.53**	**82.82**	**84.54**	**11.97**	**11.73**	**13.21**	**55.6**	**56.55**	**58.02**	**7.71**	**7.94**	**8.1**
16798	**67.28**	**64.6**	**62.98**	**41.36**	**38.75**	**35.33**	**3.93**	**2.94**	**2.74**	**82.59**	**81.54**	**82.23**	**12.37**	**7.82**	**11.98**	**55.11**	**54.8**	**58.02**	**7.66**	**7.73**	**8.1**

16799	68.45	63.66	63.03	41.92	36.77	33.86	4.09	2.94	2.83	83.41	82.72	83.09	11.12	10.66	13.49	54.37	55.38	58.02	7.6	7.75	8.07
16800	67.64	64.15	63.13	41.67	39.35	35.85	3.99	2.89	2.83	83.03	81.25	81.92	11.73	8.64	11.27	55.3	54.79	57.36	7.68	7.72	8
16801	67.26	63.72	63.86	42.16	38.68	35.69	3.99	2.84	2.74	82.49	80.97	81.95	11.41	8.98	11.5	54.54	54.93	57.28	7.58	7.75	8.05
16802	68.22	64.35	63.1	42.41	37.95	35.61	3.73	2.61	2.47	82.39	81.02	80.59	11.67	8.73	12.21	54.72	55.14	58.05	7.6	7.77	8.06
16803	67.16	65.59	63.38	40.87	37.28	34.3	3.83	2.72	2.56	82.57	83.1	82.99	11.91	10.12	12.96	55.13	56.17	58.34	7.65	7.92	8.12
16804	66.42	63.74	63.98	40.51	36.84	34.63	3.7	2.71	2.67	81.97	81.99	82.36	12.65	12.39	14.49	55.65	57.23	59.39	7.69	8.04	8.27
16805	64.97	64.03	64.22	38.2	35.46	34.65	3.7	2.7	2.68	82.17	83.15	85.54	13.75	12.48	13.69	56.82	57	57.74	7.81	8	8.02
16806	67.21	64.9	64.24	40.76	37.85	36.41	3.69	2.66	2.54	82.07	82.63	82.43	11.67	10.14	11.6	55.16	55.72	57.07	7.64	7.88	7.99
16807	65.41	60.85	59.51	40.35	36.73	34.97	3.63	2.68	2.55	81.53	79.9	79.35	12.22	12.78	14.9	55.61	56.97	58.84	7.66	7.86	8.01
16808	68.54	62.81	62.41	41.74	37.85	34.54	4.01	2.86	2.75	83.18	80.52	81.58	11.94	10.99	14.62	55.01	55.93	59.4	7.64	7.79	8.2
16809	68.54	62.53	62.39	41.89	37.48	35.88	3.98	2.83	2.78	82.65	80.64	80.89	11.41	11.16	12.84	54.31	56.73	58.04	7.55	7.91	8.01
16810	68.54	63.01	62.34	42.11	36.7	36.35	3.93	2.8	2.75	83.05	81.38	80.02	11.86	12.11	12.22	54.52	56.79	57.02	7.58	7.95	7.89
16811	66.13	62.68	62.48	40.34	36.76	34.51	3.68	2.91	2.78	82.3	81.44	81.6	13.63	13.36	14.72	56.31	58.28	59.59	7.77	8.12	8.26
16812	66.41	63.54	63.17	40.52	35.28	33.93	3.85	2.65	2.58	82.77	83.06	82.52	12.54	14.52	16.22	55.57	58.96	60.16	7.71	8.22	8.31
16813	65.49	62.13	61.42	39.43	37.52	35.05	3.57	2.79	2.66	81.74	79.6	79.83	13.13	12.37	14.42	56.57	56.65	58.8	7.77	7.86	8.07
16814	68.36	65.63	64.01	42.51	37.22	31.68	4	2.9	2.7	82.63	82.6	83.76	11.02	9.54	14.7	54.33	54.74	60.14	7.55	7.77	8.47
16815	66.31	64.2	63.74	40.89	36.04	33.73	3.93	2.73	2.61	83.07	83.47	83.03	13.24	13.2	15.57	55.84	58.71	60.33	7.72	8.26	8.37
16816	66.96	63.92	62.87	40.26	37.67	33.56	3.82	2.8	2.57	82.31	82.21	83.16	12.26	11.19	15.77	55.66	56.31	60.5	7.68	7.87	8.4
16817	67.59	64.38	63.72	41.61	36.84	32.87	3.86	2.81	2.59	82.21	82.11	82.8	11.42	10.38	13.56	54.74	56.09	58.63	7.57	7.91	8.17
16818	68.26	61.83	62.27	41.49	38.25	34.38	3.99	2.78	2.76	82.96	79.22	81.5	11.5	10.99	14.26	55.05	55.89	59.25	7.65	7.77	8.23
16819	67.81	64.58	62.41	41.86	37.98	35.68	3.84	2.8	2.74	82.77	82.43	81.56	11.7	9.3	12.04	55.19	56.64	58.2	7.69	8.01	8.11
16821	68.24	62.36	61.8	41.32	38.04	34.59	3.93	2.84	2.83	83.1	80.1	81.21	11.54	10.8	14.16	54.99	55.74	59.05	7.64	7.78	8.2
16822	69.15	63.02	62.24	41.67	37.48	34.3	4.04	2.89	2.73	83.28	80.64	81.52	10.6	10.61	14.13	54.23	55.91	59.36	7.6	7.79	8.23
16834	67.68	63.31	63.18	40.04	36.54	33.43	3.67	2.77	2.69	82.49	82.65	82.94	12.18	12.44	15.32	55.44	57.31	59.69	7.67	8.02	8.28
16835	67.15	62.68	62.1	40.8	36.98	34.14	3.74	2.8	2.68	82.73	80.73	81.19	12.19	11.66	13.91	55.06	56.68	58.5	7.64	7.9	8.1
16836	67.12	64.63	63.66	40.44	36.8	32.76	3.84	2.55	2.37	82.65	82.94	83.19	12.78	11.5	15.18	55.94	56.4	59.52	7.74	7.93	8.25
16837	68.61	63.81	62.96	42.05	37.02	33.78	4.09	2.92	2.72	83.36	82.71	82.92	11.07	11.28	14.25	54.22	55.91	58.32	7.57	7.87	8.14
16838	68.02	63.41	62.69	41.22	37.88	36.34	3.89	2.76	2.72	82.66	81.17	82.53	11.71	11.27	12.47	55.29	56.13	57.98	7.65	7.85	8.03
16839	67.13	62.08	62.21	42.04	38.82	35.42	3.88	3.01	2.86	82.64	80	81.2	11.38	11.44	13.92	54.42	56.13	58.68	7.58	7.76	8.11
16840	68.16	63.13	62.35	42.19	37.28	34.11	4.06	2.97	2.83	83.59	82.3	82.47	12.05	9.93	12.87	54.81	55.43	58.23	7.63	7.79	8.11

16902	67.14	63.03	62.76	40.89	40.13	35.72	3.87	3	2.84	82.1	80.77	81.87	12.56	9.92	14.18	55.67	55.72	59.06	7.66	7.83	8.22
18438	68.22	61.06	60.58	41.17	38.56	33.03	3.75	2.7	2.48	82.52	79.53	81.93	12.82	11.23	17.13	55.75	55.08	59.97	7.71	7.59	8.24
18448	67.53	61.49	61.05	41.64	36.4	31.57	3.86	2.66	2.44	82.43	81	82.52	12.57	12.67	18.47	55.34	57.22	62.3	7.63	7.98	8.6
18662	66.9	66.71	61.65	38.15	36.26	28.66	3.67	2.82	2.7	82.95	84.46	85.42	13.49	10.05	18.34	56.44	55.09	63.8	7.8	7.79	8.94
BAGCE 100	70.24	63.32	62.62	43.28	38.34	36.26	4.28	2.77	2.7	83.61	80.9	80.61	10.58	8.66	12.04	53.35	54.48	57.13	7.44	7.59	7.86
BAGCE 17	65.98	61.35	60.48	40.05	35.8	33.29	3.65	2.62	2.5	81.69	80.52	81.34	13.3	13.9	16.32	56.17	57.76	59.94	7.72	7.98	8.24
BAGCE 30	69.39	63.49	63.21	42.42	38.27	34.6	3.98	2.94	2.84	83.26	80.57	82.48	12.02	10.73	13.81	54.44	55.85	58.55	7.59	7.77	8.15
BAGCE 34	69.32	63.99	63.13	42.04	38.22	34.37	4.11	3.03	2.74	82.78	81.27	82.19	11.06	9.74	14.04	53.91	54.69	58.26	7.49	7.69	8.11
BAGCE 53	68.64	65.08	63.59	41.75	37.37	33.96	3.92	2.86	2.71	83.03	82.98	82.79	12.23	11.37	14.42	55.13	56.66	59.38	7.66	7.97	8.26
BAGCE 81	67.27	63.51	63.01	41.39	35.86	33.32	3.66	2.62	2.48	82.27	82.26	82.42	12.16	12.95	15.89	55.35	58.2	60.66	7.67	8.16	8.42
BAGCE 86	67.3	62.52	62.58	40.36	36.21	34.52	3.65	2.71	2.57	82.61	81.51	83.02	11.86	12.13	12.85	55.74	57.82	58.42	7.74	8.12	8.14
BAGCE 93	68.6	64.46	63.6	41.97	36.93	33.34	4.08	2.82	2.61	83.42	82.15	83.12	12.16	12.84	16.8	54.99	57.17	60.53	7.63	7.98	8.37
BAGCE 97	68.25	63.81	62.75	41.27	37.59	33.84	3.79	2.75	2.68	83.09	81.84	82.46	12.05	13.46	17.53	55.21	57.86	61.44	7.67	8.05	8.47
CNPGL 00-1-1	65.76	62.07	61.45	39.61	35.68	34.2	3.62	2.66	2.55	81.76	80.61	80.63	12.8	12.53	13.72	56.46	58.38	59.18	7.8	8.14	8.19
CNPGL 92-133-3	65.5	62.54	61.08	40.55	35.74	32.63	4.12	3.07	2.73	81.58	80.58	81.15	12.22	12.02	16.24	54.79	57.78	60.7	7.58	8	8.32
CNPGL 92-198-7	68.66	64.01	63.39	42.02	38.61	34.02	3.98	2.82	2.65	83.04	81.38	82.78	11.97	10.73	15.82	55.04	55.55	59.74	7.63	7.76	8.28
CNPGL 92-56-2	67.33	64.2	63.24	41.35	38.76	34.1	3.91	2.93	2.72	82.72	81.68	81.89	11.78	10.2	14.61	55.12	56.54	60.12	7.66	7.93	8.33
CNPGL 92-66-3	68.92	64.49	63.48	42.14	38.22	34.84	3.88	2.75	2.59	83	81.71	81.9	11.92	9.41	12.35	54.44	55.53	58.39	7.57	7.79	8.12
CNPGL 9279-2	67.52	64.76	63.62	41.65	39.12	35.5	3.86	2.83	2.71	82.26	81.59	82.3	11.76	10.5	13.72	54.76	56.01	58.7	7.6	7.85	8.17
CNPGL 93 -37-5	66.7	63.48	62.77	39.7	36.81	34.53	3.77	2.9	2.72	81.97	81.98	82.22	13.25	12.64	14.87	55.71	57.99	59.17	7.69	8.12	8.22
CNPGL 93-01-1	67.35	62.36	60.11	42.05	37.55	35.63	4	2.99	2.81	81.98	79.69	78.55	12.17	12.94	15.01	55.14	57.94	59.04	7.61	8.01	8.02
CNPGL 93-04-2	65.71	63.49	61.46	40.65	37.2	35.92	3.83	3.1	2.86	81.83	81.39	79.89	12.49	12.25	13.19	55.34	57.09	57.89	7.62	7.95	7.93
CNPGL 93-18-2	67.22	64.86	63.67	42.37	39.96	35.96	4.05	3.06	2.9	82.73	81.51	81.88	11.68	8.71	12.81	54.8	54.68	57.79	7.62	7.68	8.03
CNPGL 94-13-1	67.27	65.08	64.56	41.55	37.52	37.07	4.01	3	2.93	82.24	83.07	82.69	12.6	12.3	12.18	55.14	57.45	57.45	7.6	8.07	8.02

CNPGL 96-21-1	66.55	64.66	62.99	40.6	36.27	34.83	3.87	2.88	2.72	82.03	82.06	81.52	12.66	11.66	13.61	55.41	56.79	57.95	7.68	7.95	8.04
CNPGL 96-23-1	67.05	63.92	63.2	40.69	35.58	34.67	3.69	2.69	2.63	82.48	83.24	82.99	12.51	11.86	13.85	55.85	57.77	58.56	7.73	8.16	8.13
CNPGL 96-27-3	66.67	64.03	63.16	40.91	37.85	36.1	3.94	3	2.94	82.01	82.23	81.92	11.98	10.72	12.16	55.19	56.07	57.58	7.65	7.89	8.03
PIONEIRO	67.12	63.37	62.89	40.15	35.18	32.72	3.64	2.71	2.58	82.87	82.54	83.14	12.39	12.05	14.73	56.28	57.83	59.81	7.83	8.19	8.39
LSD(5%)	0.75	0.74	0.27	0.57	0.96	0.97	0.1	0.04	0.04	0.3	0.27	0.73	0.61	0.68	0.67	0.52	0.47	0.46	0.07	0.12	0.12

Table S3: Mean genotype values for Leaf and stem tissue samples for feed quality traits under Wet growing condition.

	NDF		ADF		ADL		OM		CP		IVOMD		Me	
Genotype/Trait	Leaf	Stem	Leaf	Stem	Leaf	Stem	Leaf	Stem	Leaf	Stem	Leaf	Stem	Leaf	Stem
1026	67.69	72.99	41.29	47.14	4.2	4.64	82.92	85.87	11.7	8.36	54.49	52.56	7.53	7.49
14355	67.77	68.5	41.5	44.25	4.07	4.1	82.92	85	11.95	9.89	55.08	55.21	7.6	7.76
14389	66.31	69.94	41.67	44.61	4.13	3.56	81.59	85.32	12.07	10.22	54.71	54.02	7.51	7.61
14982	66.06	70.9	41.78	45.49	4.19	3.56	81.01	84.53	11.54	10.26	54.19	54.47	7.44	7.66
14983	66.1	71.87	40.98	46.19	4.19	4.01	81.82	84.72	13.4	8.98	55.98	54.27	7.67	7.68
14984	67.07	68.23	42.25	44.38	4.21	3.61	82.22	84.72	11.78	12.25	54.71	57.19	7.56	8.02
15357	68.23	68.28	41.73	44.36	4.12	3.98	83.12	85.25	12.12	10.03	55.01	55.85	7.62	7.88
15743	67.84	73.06	42.78	48.94	4.14	4.05	82.24	84.03	11.46	8.25	54.43	52.69	7.54	7.44
16621	67.37	75.3	39.87	45.67	4.24	5.03	84.06	88.76	14.69	11.08	55.67	55.12	7.68	7.77
16782	67.43	72.44	41.44	47.18	4.09	4.04	82.66	85.02	11.61	8.24	54.75	53.21	7.58	7.56
16783	68.02	70.11	42.94	46.52	4.06	3.9	82.3	83.63	11.19	10.93	54.19	54.73	7.51	7.66
16784	65.74	69.43	41.28	45.32	4.14	3.86	81.3	84.38	11.81	9.85	54.81	55.09	7.55	7.77
16785	67.72	69.51	41.45	44.08	4.01	3.08	82.04	85.08	11.41	11.22	54.81	55.41	7.59	7.79
16786	69.59	72.86	43.53	48.78	4.57	4.4	83.09	84.67	10.67	9.12	53.35	52.91	7.44	7.5
16787	67	67.92	41.16	45.46	4.11	3.47	81.97	84.7	12.5	10.89	55.37	55.36	7.64	7.77

16788	68.01	71.49	42.34	47.2	4.18	3.93	82.23	83.67	12.04	10.56	54.71	54.76	7.56	7.66
16789	67.5	68.62	42.5	44.46	4.29	3.48	82.07	84.48	11.4	11.61	54.21	56.32	7.5	7.89
16790	64.89	71.86	39.84	46.76	3.68	3.92	80.93	84.01	12.63	8.62	55.75	53.31	7.66	7.52
16791	67.77	70.88	42.09	46.3	4.07	3.84	82.02	83.58	11.65	10.5	54.75	54.95	7.57	7.69
16792	68.1	69.52	42.81	45.03	4.26	3.53	82.42	85.56	10.58	10.35	53.85	55.54	7.48	7.86
16793	69.22	71.09	41.81	45.2	4.14	3.69	83.34	84.85	11.55	9.65	54.48	55.16	7.56	7.81
16794	66.72	74.12	42.23	49.92	4.5	4.8	81.58	83.91	12.58	9.78	54.25	51.43	7.45	7.2
16795	67.38	68.08	42.58	44.26	4.3	3.25	82.01	84.96	11.34	11.76	54.13	56.07	7.49	7.86
16796	68.1	74.46	41.76	47.92	4.25	4.28	82.02	84.84	11.13	8.33	54.33	52.83	7.51	7.48
16797	67.29	72.88	41.53	46.84	3.95	4.25	81.95	84.8	10.74	10.08	53.55	52.98	7.4	7.41
16798	67.64	68.94	42.59	46.29	4.32	3.95	82.47	84.63	11.77	12.06	54.55	55.75	7.54	7.77
16799	68.45	71.41	42.59	46.31	4.45	3.99	83.2	84.57	10.23	8.94	53.58	54.39	7.48	7.71
16800	67.21	68.86	42.42	44.37	4.27	3.38	81.9	85.44	11.12	11.43	54.15	56.07	7.49	7.9
16801	67.53	70.77	42.85	45.33	4.12	3.59	82.13	85.21	10.87	9.88	53.92	55.43	7.48	7.86
16802	67.98	71.64	41.91	46.28	3.99	4.03	82.2	83.98	11.88	9.67	54.91	54.27	7.58	7.67
16803	66.71	67.6	40.68	44.46	3.91	3.36	81.88	83.03	11.83	10.77	55.03	54.36	7.6	7.55
16804	66.58	68.16	41.39	45.22	4.06	3.68	81.67	83.07	11.76	10.81	54.71	54.64	7.56	7.59
16805	65.77	68.64	39.12	45.58	4.58	4.39	81.08	80.93	12.86	10.37	55.82	55.07	7.64	7.6
16806	66.18	67.89	41.76	42.73	4.16	2.83	81.18	84.17	11.87	12.47	54.64	56.85	7.52	7.95
16807	65.95	70.38	40.67	44.63	3.97	3.56	81.56	83.1	11.86	10.28	54.88	54.43	7.54	7.6
16808	67.75	73.99	41.77	48.12	4.31	4.19	82.31	85.07	11.64	8.08	54.65	52.33	7.55	7.44
16809	68.36	72.82	42.32	46.9	4.3	3.91	82.37	84.62	10.7	8.52	53.72	53.73	7.44	7.6
16810	67.14	74.24	41.36	48.14	4.22	4.45	81.98	85.19	12.4	8.58	54.8	52.5	7.54	7.43
16811	65.73	70.21	40.33	45.55	4.06	3.99	81.95	84.94	13.15	10.93	56.02	55.74	7.72	7.85
16812	66.34	66.91	40.51	43.03	4.19	3.68	82.84	83.72	12.32	12.62	55.19	57.06	7.63	7.92
16813	65.28	67.85	39.59	42.63	4.04	3.57	81.35	85.41	13.03	11.08	56.15	56.66	7.71	8.02
16814	68.03	73.44	42.04	48.47	4.12	4.43	82.21	83.97	11.12	8.6	54.01	52.48	7.46	7.4
16815	67.32	68.97	41.71	44.33	4.24	3.84	83.16	84.13	12.17	11.47	54.84	55.87	7.61	7.84
16816	65.94	72.06	41.04	46.22	3.96	3.85	81.7	84.39	12.53	11.96	55.11	54.67	7.55	7.61
16817	67.76	70.25	41.73	46.14	4.1	4.01	82.38	83.72	11.39	9.96	54.35	53.75	7.53	7.54

16818	67.79	73.96	41.38	48.01	4.2	4.29	82.34	85.06	11.44	7.6	54.37	52.83	7.51	7.51
16819	66.96	69.44	41.62	44.67	4.04	3.41	82.11	84.98	12	11.4	55.27	56.65	7.66	8
16821	67.23	73.88	41.23	48.14	4.09	4.41	82.18	85.63	11.48	7.42	54.59	52.57	7.56	7.51
16822	68.49	74.35	41.77	47.66	4.22	4.35	82.71	85.86	10.52	7.42	54.07	52.05	7.51	7.43
16834	66.7	72.65	40.45	46.24	3.95	3.82	82.14	84.74	11.78	10.04	54.82	53.58	7.58	7.55
16835	66.96	70.8	41.38	45.64	4.11	4.04	82.59	84.53	11.98	10.37	54.86	54.34	7.57	7.63
16836	67.69	70.28	41.68	44.88	4.23	3.58	82.05	84.68	11.46	9.74	54.74	56.2	7.58	7.96
16837	68.62	72.15	42.53	46.9	4.42	3.98	83.03	84.56	10.77	8.61	53.6	53.6	7.45	7.59
16838	67.38	74.58	41.54	47.46	4.09	4.08	81.81	85.21	11.2	9.83	54.38	53.88	7.51	7.58
16839	66.98	70.08	42.26	45.2	4.24	3.84	81.77	84.58	11.21	8.56	53.88	53.75	7.45	7.64
16840	67.76	72.36	42.63	47.45	4.38	4.12	83.38	85.09	11.9	10.3	54.44	54.53	7.54	7.7
16902	67.14	67.96	42.06	45.3	4.31	3.56	81.31	83.32	11.76	10.99	54.53	53.37	7.5	7.42
18438	67.84	71.98	42.28	46.64	4.12	4.1	82.31	83.78	12.01	10.84	54.75	54.66	7.53	7.62
18448	66.51	70.18	41.83	46.59	4.13	3.61	81.64	83.26	11.67	8.93	54.54	52.78	7.48	7.37
18662	73.46	76.66	41.83	49.94	5.88	5.44	80.69	84.53	12.26	7.54	55.83	53.08	7.7	7.54
BAGCE 100	68.15	75.22	42.24	50.25	4.34	5.44	82.1	86.03	11.2	6.87	53.75	50.13	7.41	7.13
BAGCE 17	65.82	67.55	41	43.14	4	3.42	81.52	81.34	13.04	12.47	55.5	56.53	7.59	7.76
BAGCE 30	68.54	73.52	42.69	48.33	4.29	4.43	82.7	84.7	11.54	8.69	54	53.02	7.47	7.51
BAGCE 34	68.18	73.44	41.98	47.35	4.36	4.44	81.96	84.7	11.27	9.2	53.68	52.94	7.42	7.47
BAGCE 53	67.61	74.13	41.03	48.43	4.01	4.49	82.71	85.9	12.4	8.13	55.45	52.71	7.66	7.52
BAGCE 81	66.46	69.93	41.35	45.04	3.96	3.93	81.5	83.74	11.99	10.12	54.78	55.29	7.55	7.8
BAGCE 86	65.2	72.05	40.02	46.26	3.81	3.9	81.6	85.31	12.03	7.71	55.52	53.22	7.67	7.6
BAGCE 93	67.75	71.91	42.01	47.06	4.34	4.41	82.69	85.56	12.33	9.72	54.69	53.56	7.57	7.57
BAGCE 97	67.1	72.91	41.31	46.48	4.13	3.93	82.35	84.41	12.07	9.46	55.08	54.04	7.59	7.6
CNPGL 00-1-1	64.94	69.8	40.33	45	3.9	3.69	80.97	83.95	12.66	10.6	55.98	56.43	7.68	7.93
CNPGL 92-133-3	65.18	70.14	40.64	46.58	4.24	4.39	81.02	83.99	12.38	9.37	54.64	53.64	7.48	7.55
CNPGL 92-198-7	67.29	70.62	41.99	47.09	4.21	4.5	82.24	84.19	12.11	10.07	54.69	54.12	7.54	7.59
CNPGL 92-56-2	66.19	66.19	41.35	43.34	4.17	3.9	81.69	84.47	12.02	10.91	54.86	56.57	7.57	7.96

**CNPGL 92-66-3**	**67.87**	**72.9**	**42.52**	**47.65**	**4.14**	**3.85**	**82.2**	**84.49**	**11.22**	**9.59**	**54.15**	**54.72**	**7.49**	**7.72**
**CNPGL 9279-2**	**67.99**	**70.81**	**42.16**	**46.69**	**4.24**	**4.12**	**82.19**	**83.92**	**11.29**	**9.4**	**54.36**	**54.13**	**7.53**	**7.63**
**CNPGL 93-01-1**	**66.14**	**69.72**	**40.26**	**45.12**	**4.08**	**3.71**	**81.89**	**83.51**	**12.94**	**10.27**	**55.7**	**55.02**	**7.66**	**7.69**
**CNPGL 93-04-2**	**65.85**	**72.58**	**41.92**	**47.68**	**4.28**	**4.31**	**81.02**	**85.31**	**12.58**	**9.39**	**55.1**	**53.99**	**7.54**	**7.64**
**CNPGL 93-18-2**	**65.8**	**68.42**	**41.24**	**45.03**	**4.22**	**4.02**	**81.13**	**83.48**	**12.05**	**9.31**	**54.6**	**54.65**	**7.5**	**7.69**
**CNPGL 93 -37-5**	**66.58**	**72.02**	**42.68**	**47.52**	**4.17**	**4.26**	**81.73**	**85.43**	**11.42**	**9.48**	**54.36**	**54.27**	**7.51**	**7.7**
**CNPGL 94-13-1**	**67.71**	**71.42**	**42.31**	**48.58**	**4.39**	**4.51**	**81.98**	**83.06**	**12.17**	**9.87**	**54.49**	**53.44**	**7.5**	**7.43**
**CNPGL 96-21-1**	**65.06**	**70.74**	**40.62**	**45.28**	**4.15**	**3.89**	**81.16**	**84.88**	**12.59**	**10.72**	**55.35**	**55.24**	**7.59**	**7.77**
**CNPGL 96-23-1**	**65.52**	**72.04**	**39.95**	**48**	**3.8**	**4.27**	**81.84**	**85.18**	**12.7**	**7.69**	**55.87**	**52.72**	**7.71**	**7.47**
**CNPGL 96-27-3**	**65.51**	**69.75**	**41.2**	**46.06**	**4.16**	**4.11**	**81.39**	**84.71**	**11.9**	**9.37**	**54.85**	**54.2**	**7.56**	**7.66**
**PIONEIRO**	**65.8**	**71.33**	**40.38**	**45.37**	**3.91**	**3.95**	**81.72**	**84.91**	**12.41**	**9.4**	**55.85**	**54.8**	**7.69**	**7.76**
**LSD (5%)**	**2.92**	**4.78**	**2.39**	**3.64**	**0.51**	**0.78**	**2.1**	**1.65**	**4.64**	**3.36**	**5.55**	**3.48**	**0.9**	**0.46**
